# A Systematic Review of Extracellular Matrix-Related Alterations in Parkinson’s Disease

**DOI:** 10.3390/brainsci14060522

**Published:** 2024-05-21

**Authors:** Mary Ann Chapman, Barbara A. Sorg

**Affiliations:** 1 Visage Communications, Spokane, WA 99021, USA; 2 R.S. Dow Neurobiology, Legacy Research Institute, Portland, OR 97232, USA; bsorg@downeurobiology.org

**Keywords:** collagen, extracellular matrix, focal adhesion, cell adhesion, proteoglycan, glycosaminoglycan, matrisome

## Abstract

The role of the extracellular matrix (ECM) in Parkinson’s disease (PD) is not well understood, even though it is critical for neuronal structure and signaling. This systematic review identified the top deregulated ECM-related pathways in studies that used gene set enrichment analyses (GSEA) to document transcriptomic, proteomic, or genomic alterations in PD. PubMed and Google scholar were searched for transcriptomics, proteomics, or genomics studies that employed GSEA on data from PD tissues or cells and reported ECM-related pathways among the top-10 most enriched versus controls. Twenty-seven studies were included, two of which used multiple omics analyses. Transcriptomics and proteomics studies were conducted on a variety of tissue and cell types. Of the 17 transcriptomics studies (16 data sets), 13 identified one or more adhesion pathways in the top-10 deregulated gene sets or pathways, primarily related to cell adhesion and focal adhesion. Among the 8 proteomics studies, 5 identified altered overarching ECM gene sets or pathways among the top 10. Among the 4 genomics studies, 3 identified focal adhesion pathways among the top 10. The findings summarized here suggest that ECM organization/structure and cell adhesion (particularly focal adhesion) are altered in PD and should be the focus of future studies.

## 1. Introduction

The extracellular matrix (ECM) is a structural and functional complex that surrounds and anchors nearly all cells in the body. The core matrisome consists of approximately 300 proteins that have been grouped broadly into collagens, glycoproteins, and proteoglycans [1]. The presence and ratio of these proteins vary by organ and tissue type [1,2]. Compared with other organs, the brain is relatively poor in fibrous proteins such as collagen but rich in proteoglycans that contain negatively charged glycosaminoglycan (GAG) side chains that sequester cations and water.

Beyond giving the brain its gelatinous texture, the ECM plays numerous functional roles. Through its binding to integrins in cell membranes and its downstream effects on the cytoskeleton, the ECM signals cell survival and maintains differentiation, polarity, and morphology [3,4]. The ECM also regulates growth factor function through binding and releasing growth factor proteins and their fragments. For example, the ECM component heparan sulfate serves as a low-affinity binding site for basic fibroblast growth factor (bFGF), which is essential for its high-affinity binding [5]. Signaling by glial cell line-derived neurotrophic factor (GDNF) requires heparan sulfate glycosaminoglycan [6], and the heparan sulfate proteoglycan syndecan-3 is a novel receptor for GDNF and other GDNF family ligands that mediate cell spreading and neurite outgrowth [7].

Another key function of the ECM is in the formation and plasticity of synapses. Many recent plasticity investigations have centered on perineuronal nets, ECM structures comprising chondroitin sulfate proteoglycans, link proteins, hyaluronic acid, and tenascins that surround the selected somas and proximal dendrites [8,9]. Perineuronal nets appear during the critical period of synaptic development and help stabilize synapses, although they alter plasticity, even in adulthood [10,11]. This plasticity has been demonstrated in various neuronal systems and models through the digestion of chondroitin sulfate, which reactivates ocular dominance plasticity in adult rats [12] and restores memory in mice with tau pathology [13]. However, the function of perineuronal nets on plasticity may be more complex, as digestion of perineuronal nets can also impair learning and memory [10]. Other ECM arrangements are also found in the nervous system, including diffuse ECM containing axonal coats [14] and perinodal ECM [15], although their roles have been studied less. 

As an integral component of brain tissue that dynamically interacts with neurons and glia, the ECM is expected to be affected by, and contribute to, neurodegenerative processes in diseases such as Parkinson’s disease (PD) and Alzheimer’s disease (AD). Indeed, over the past decade, evidence has accumulated that implicates the ECM in neurodegeneration [16] and PD specifically [17,18]. Studies that find such effects have tended to analyze transcriptomics, proteomics, and genomics data using pathway approaches or gene sets instead of individual protein or gene comparisons. Pathway analyses permit the assessment of cumulative changes throughout individual biological processes that may, together, exert an effect on cell or tissue structure and function. This systematic review was, therefore, designed to identify the top deregulated ECM-related pathways in studies that used gene set enrichment analysis to document transcriptomic, proteomic, or genomic alterations in PD.

## 2. Materials and Methods

The PubMed database was searched for research reports using the following search strategy: Parkinson’s disease AND (proteomics OR GWAS OR gene expression OR RNA sequencing analysis OR enrichment OR pathways OR KEGG OR gene ontology OR Reactome) AND (extracellular matrix OR adhesion OR collagen OR proteoglycans OR glycosaminoglycans). Given that searches in Google Scholar are organized differently from those in PubMed, the following advanced search strategy was used in Google Scholar: Parkinson disease (all of the words), extracellular matrix (exact phrase), and at least one of the following words, where the words were listed anywhere in the article: KEGG OR ontology OR Reactome. The results were sorted by relevance. The title and/or abstract of each result was read to identify relevant studies, and the reference lists of studies and review articles were consulted for additional articles that the searches missed. This study was not registered on Prospero.

Articles were included if they (1) described published, peer-reviewed research studies of transcriptomics, proteomics, or genomics in PD, (2) were conducted using tissues or cells derived from humans with PD, (3) employed gene set enrichment analysis (GSEA), and (4) identified one or more ECM-related pathway among the top-10 enriched gene sets. Reviews, preprints, and studies that evaluated ECM in animal models of PD were excluded, although very few studies have reported changes in the ECM in animal models of PD [19,20,21], with the major focus on matrix metalloproteinases (MMPs), which regulate the ECM [22,23,24]; for review, see [25]. Studies conducted in multiple disease populations were also excluded unless results for Parkinson’s disease patients were reported separately. 

Articles that met the criteria were reviewed, and study information was extracted into a summary table. Gene sets considered ECM-related were adhesion groups or pathways (adherens junctions, cell adhesion, cell adhesion molecules, cell adhesion substrate, and focal adhesions) and those that had ECM, collagen, integrin, GAG, or proteoglycan in the pathway name (e.g., ECM organization). ECM-related gene sets or pathways reported by the authors of each study to be among the top-10 deregulated or enriched (as defined by the study authors) were recorded in the summary table.

## 3. Results

**Figure 1:**
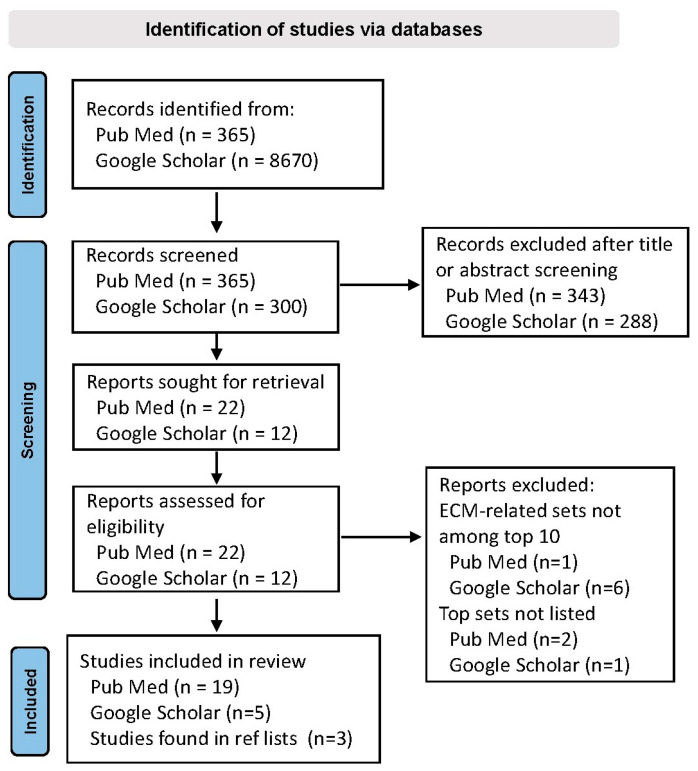
Preferred Reporting Items for Systematic Reviews and Meta Analyses (PRISMA) flow diagram outlining the search strategy.

The PubMed search yielded 365 results, 19 of which met the inclusion criteria. The Google Scholar search yielded 8670 results; after the first 300 results in the Google Scholar search, relevance declined dramatically, and, thus, the remaining pages were not considered. Five additional articles identified in the Google Scholar search but not in the PubMed search met the criteria. Searches of the reference lists of relevant articles identified 3 additional studies with prominent ECM findings, for a total of 27 studies (Figure 1). All studies included one or more control groups.

Studies could be classified as transcriptomics, proteomics, or genomics; two studies used multiple omics analyses and are, therefore, entered into the tables twice (in different sections) (Table 1, Supplemental Table S1). The gene set enrichment analyses were conducted using various databases, such as Kyoto Encyclopedia of Genes and Genomes (KEGG), Reactome, and gene ontology (GO) terms. Top pathways were identified by the authors of each article based on *p* values adjusted for multiple testing or q values.

### 3.1. Transcriptomics Studies

**Figure 2:**
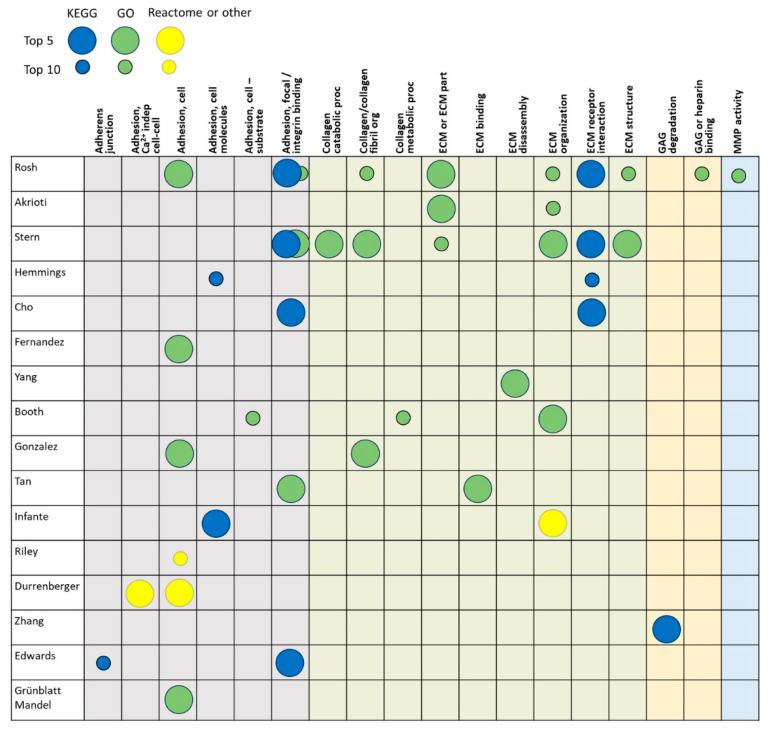
Transcriptomic studies: Top deregulated ECM-related gene sets/pathways. Columns are grouped by adhesion (gray), ECM or collagen (light green), GAG related (light yellow), and MMP related (light blue). Large circles indicate that the gene set or pathway was among the top 5 and smaller circles indicate that the gene set or pathway was among the top 10, as defined by individual study authors. Dark-blue circles denote KEGG pathways, green circles denote GO gene sets, and yellow circles denote Reactome or other pathways [17,26,27,28,29,30,31,32,33,34,35,36,37,38,39,40,41]. Abbreviations: ECM = extracellular matrix, GAG = glycosaminoglycan, GO = gene ontology, KEGG = Kyoto Encyclopedia of Genes and Genomes, MMP = matrix metalloprotease, org = organization, proc = process.

The 17 transcriptomics studies (16 data sets) identified in the search were conducted on a variety of human cell and tissue types and included both monogenic and idiopathic PD (Table 1 and Supplemental Table S1) [17,26,27,28,29,30,31,32,33,34,35,36,37,38,39,40,41]. Most of the studies performed gene set enrichment analysis using GO terms or KEGG pathways, but three used Reactome, Metabase Ontology, or Pathway Studio (Figure 2; Supplemental Table S1). The studies assessed deregulated pathways in induced pluripotent stem cells (iPSCs) [26,27,28,32], dermal fibroblasts [17,33], mesenchymal stem cells [30], blood [29,31,34,35], and various brain regions [36,37,38,39,40,41].

Thirteen of the sixteen data sets identified one or more adhesion pathways in the top-10 deregulated gene sets or pathways (Figure 2). These were primarily related to cell adhesion (8 of 16 data sets) and focal adhesion/integrin binding (5 of 16 data sets). Four studies identified altered collagen gene sets or pathways, and nine identified overarching ECM gene sets or pathways among the top 10. One study reported somewhat different results for iPSC cell lines from those with sporadic PD versus PD associated with GBA1 mutations [26].

### 3.2. Proteomics Studies

The eight proteomics studies identified in the search were also conducted on a variety of human cell and tissue types and included monogenic and idiopathic PD (Table 1 and Supplemental Table S1) [18,36,42,43,44,45,46,47]. These studies identified deregulated collagen proteins or collagen pathways in iPSCs [42], cerebrospinal fluid [45] tears [44], plasma [46], serum exosomes [47], and various brain regions [18,36,43].

**Figure 3:**
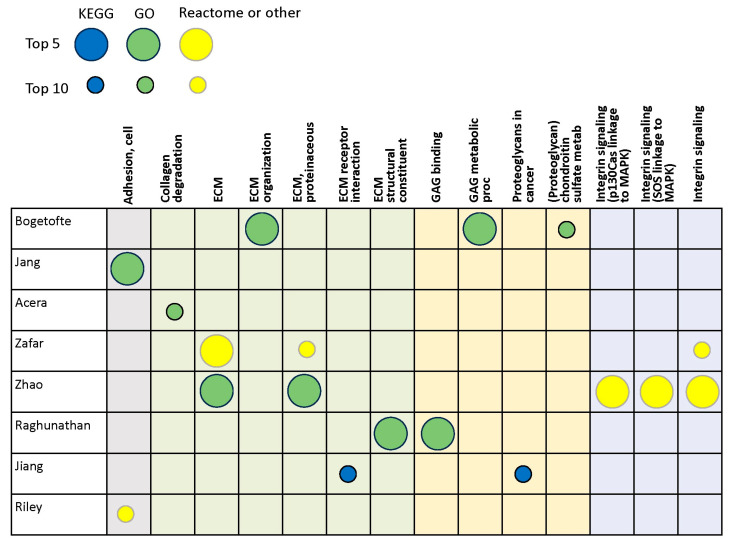
Proteomics studies: Top deregulated ECM-related gene sets/pathways. Columns are grouped by adhesion (gray), ECM or collagen (light green), GAG-proteoglycan related (light yellow), and integrin signaling (light blue). Large circles indicate gene sets or pathways among the top 5, and small circles among the top 10, as defined by individual study authors. Dark-blue circles denote KEGG pathways, green circles denote GO gene sets, and yellow circles denote Reactome or other pathways [18,36,42,43,44,45,46,47]. Abbreviations: ECM = extracellular matrix, GAG = glycosaminoglycan, GO = gene ontology, KEGG = Kyoto Encyclopedia of Genes and Genomes, metab = metabolism, proc = process.

Only two of the proteomics studies identified an adhesion pathway among the top-10 deregulated pathways [36,43], although another found three integrin signaling pathways among the top five [46], which are related to focal adhesions (Figure 3). Five of the eight proteomics studies identified altered overarching ECM gene sets or pathways among the top 10, and three of the six identified GAG or proteoglycan pathways among the top 10 (Figure 3).

### 3.3. Genomics Studies

**Figure 4:**
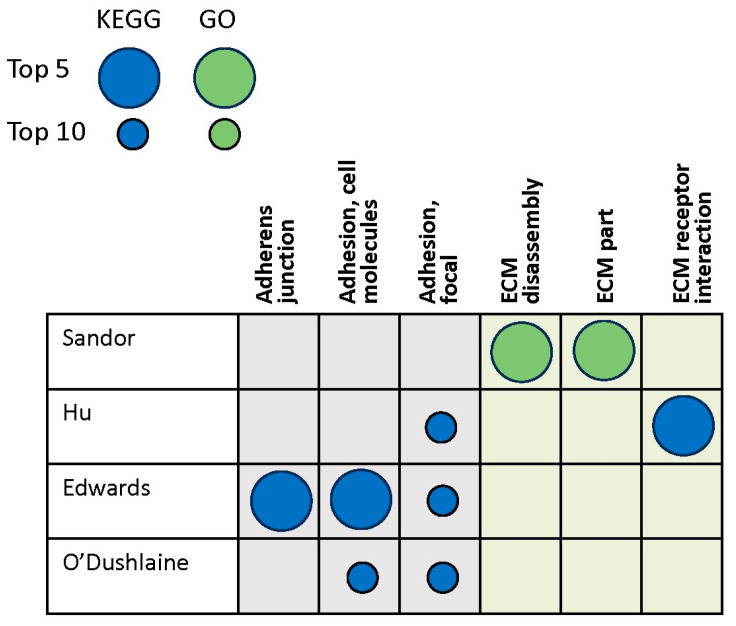
Genomics studies: Top enriched ECM-related gene sets/pathways. Columns are grouped by adhesion (gray) and ECM (light green). Large circles indicate gene sets or pathways among the top 5, and small circles among the top 10, as defined by individual study authors. Dark-blue circles denote KEGG pathways and green circles denote GO gene sets [39,48,49,50]. Abbreviations: ECM = extracellular matrix, GO = gene ontology, KEGG = Kyoto Encyclopedia of Genes and Genomes.

Four genomics studies were identified in the search, which included patients with familial and idiopathic PD (Table 1, Supplemental Table S1) [39,48,49,50]. Three of these four studies identified adhesion pathways among the top 10 most enriched, and all of them included the focal adhesion pathway (Figure 4). In the fourth study, ECM constituents and ECM disassembly were the only gene sets in the entire study identified as statistically significant [48]. The genomics studies did not identify any GAG- or proteoglycan-related gene sets among the top 10.

## 4. Discussion

This review documents the frequent alterations in ECM-related pathways reported in transcriptomics, proteomics, and genomics studies of PD. The transcriptomics and proteomics studies were conducted with different cell and tissue types, and in different brain regions, in accord with the involvement of multiple tissues and neural systems in PD and the plethora of motor and non-motor symptoms [51,52]. The studies also spanned different PD populations, including those with sporadic PD and variations in PD-related genes, such as LRRK-2, Parkin, and SNCA.

Among the different types of omics studies, ECM findings in GWAS pathway studies were the most consistent and were documented in several demographically disparate populations [39,49]. The focal adhesion pathway—dynamic protein complexes that connect the cytoskeleton to the ECM—was among the top-10 pathways in three of the four genomics studies reviewed here. These findings are supported by a recent, novel modeling approach, designed to explore disease mechanisms based on genomic data, which also identified focal adhesion as a top enriched pathway in PD [53]. Alterations in individual ECM genes have rarely been reported in PD, although scattered exceptions exist, such as the GPNMB gene [54,55,56], which is involved in cell adhesion [57]. This suggests that numerous, less frequent variations across multiple ECM genes drive the significance in the GWAS pathway analyses and may collectively constitute a risk factor for PD.

Adhesion gene sets and pathways were also among the top pathways identified in the transcriptomics studies (13 of 16 data sets) in this review. These findings are supported by those of Stern and colleagues, who have consistently reported deregulation of the focal adhesion pathway in their studies with iPSC-derived neurons [26,28,58,59], as well as in a recent systematic review of deregulated proteins in PD brains [60]. Here, we extend their findings to include studies that reported alterations in adhesion gene sets or pathways beyond the brain. For example, cell or focal adhesion alterations were noted in studies on iPSC-derived dopamine neurons and astrocytes, mesenchymal stem cells, dermal fibroblasts, whole blood, and brain. These observations suggest that ECM alterations are not necessarily restricted to areas of cell loss in the PD brain but are likely a more general disease-related phenomenon. The observation of adhesion deregulation in fibroblasts [17] is particularly interesting as fibroblasts show increased growth in PD [61] rather than degeneration.

Transcriptomic and proteomic studies included in the present review also frequently identified alterations in collagen and ECM structural and organizational gene sets/pathways. These alterations are typified by the description in the gene ontology set for ECM organization: “a process that is carried out at the cellular level which results in the assembly, arrangement of constituent parts, or disassembly of an extracellular matrix [62]”. These gene sets and pathways include proteins, such as collagens, transforming growth factor-beta (TGF-beta), matrix metalloproteinases (MMPs), and disintegrin and metalloproteases (ADAMs), as well proteins that form the basement membrane lining epithelial and endothelial tissues, such as the blood vessels. Of the few studies that have specifically assessed the ECM in PD, alterations in MMPs and basement membrane proteins are among the most frequent findings [63,64,65,66,67].

Few studies in the present review identified alterations in glycosaminoglycan (GAG) or proteoglycan gene sets/pathways. These ECM components play critical structural roles in cells and regulate cell signaling by binding and releasing growth factors [1]. However, GAGs and proteoglycans are difficult to study because of their extensive glycosylation, which is critical to their function but difficult to measure, particularly using proteomics techniques [64,65]. Novel methods may be needed to adequately explore the role of these biochemicals in PD and other diseases [64]; some of these glycomic and glycoproteomic methods are described in a recent review [68].

The results described here add to a growing literature documenting deregulation of ECM-related pathways in PD. The findings in genomics, transcriptomics, and proteomics studies conducted in non-neuronal tissues suggest that ECM-related alterations may not be a response to PD medications, which has been a concern with brain tissue. Moreover, ECM-related alterations have been identified in several studies investigating the role of alpha-synuclein [69,70], a protein frequently misfolded and aggregated in PD brains [71] and the genetic variations of which are causes and/or risk factors for PD [72]. These studies found that ECM-related pathways were among the top-5 deregulated pathways in human cell lines that overexpressed alpha synuclein [69,70]. Additional interactions among ECM molecules and alpha synuclein have been documented in animal and in vitro studies [73,74,75,76,77,78,79,80]. For instance, in a study of oligodendrocyte progenitor cells, overexpression of alpha synuclein led to integrin down-regulation that reduced adhesion to fibronectin and led to cell death [81]. Additionally, a GSEA analysis of a human cell line found that alpha synuclein overexpression led to reduced expression of ECM, collagen, and heparin gene sets [70].

Several lines of research have focused on the interactions between the ECM and extracellular alpha synuclein based on evidence that alpha synuclein can be secreted from cells via exosomes in an activity-dependent manner [82,83]. One area of investigation is the identification of mechanisms by which alpha synuclein activates microglia, initiating a proinflammatory response characterized by elevated cytokine production [84,85]. At least one of the receptors involved in microglial activation is the integrin CD11b (the alpha chain of integrin αMβ2); alpha synuclein binding to integrin CD11b activates microglial NADPH oxidase (NOX2) through a RhoA-dependent pathway, a potential mechanism by which extracellular alpha synuclein could cause neuronal damage and death [86].

Other research has focused on interactions between extracellular alpha synuclein and GAGs or proteoglycans as a potential mechanism of prion-like spreading and seeding of alpha synuclein aggregates [18], which has been hypothesized to underlie PD progression [87]. GAGs are localized to Lewy bodies in PD [88] and inhibit proteases that degrade alpha synuclein [89]. Moreover, the internalization of alpha synuclein fibrils into neuroblastoma and oligodendrocyte-like cells depends on heparan sulfate [76]. Other findings emphasize the potential protective nature of heparin in synuclein pathology. For instance, using electron microscopy, a recent in vitro study visualized alpha synuclein fibrils complexed with heparin, the presence of which altered fibril morphology and led to reduced pathological aggregation of endogenous alpha synuclein in primary cortical neurons [90].

In addition to direct interactions between the ECM and extracellular alpha synuclein, functional intracellular interactions likely occur at the synapse via the cytoskeleton. Synaptic dysfunction is an early pathological event in PD, marked by decreased striatal dopamine synthesis, storage, release, and transporter binding [91]. Wild-type alpha synuclein is concentrated at synapses, where it binds actin, slowing polymerization and accelerating depolymerization; in contrast, the PD-associated A30P mutant alpha synuclein increases actin polymerization and disrupts the cytoskeleton during the reassembly of actin filaments [92]. The ECM is a major regulator of the cytoskeletal and synaptic structure via integrin binding [93], and the focal adhesion pathway is among the most frequently dysregulated in PD [60]. Based on these observations, investigations into the interactions between ECM/focal adhesions and alpha synuclein at the synaptic cytoskeleton appear warranted.

Notably, ECM-related alterations are not specific to PD, as evidence from published omics studies that examined multiple neurodegenerative diseases has also documented the significance of ECM-related pathways, including adhesion pathways [94,95]. An analysis of 16 proteomics studies of PD, AD, and Huntington’s disease (HD) based on postmortem brain tissue found dysregulation of various biological processes across the three diseases, including ECM assembly and organization, basement membrane organization, and metabolism of GAGs and proteoglycans [65]. The only individual protein altered across all three diseases was collagen type I alpha 2, a gene expressed primarily in connective tissue but also found in the brain, where it drives glioblastoma progression [96]. Another bioinformatics study analyzed a database of differentially expressed miRNAs in body fluids and circulating cells of patients with PD, AD, amyotrophic lateral sclerosis (ALS), and multiple sclerosis (MS) from 72 studies. They found that pathways consistently targeted by several miRNAs included ECM receptor interactions, adherens junctions, and TGF-beta signaling [97]. The regulation of cell matrix adhesion was among the top protein–protein interaction pathways. An analysis of transcriptomic data from AD, PD, HD, and ALS studies found that ECM organization was among the top-8 overrepresented biological processes across the diseases, and all six detected cellular component GSEA terms related to focal adhesion, plasma membrane, and endoplasmic reticulum lumen [94]. These findings suggest that ECM-related pathways are among those broadly affected in neurodegenerative disease.

Given that dopaminergic neurons in the substantia nigra, pars compacta (SNpc) are much more susceptible to death in PD than those in the ventral tegmental area (VTA) [98], researchers have sought to identify differences in gene or protein expression that may protect VTA neurons or predispose SNpc neurons. A recent study by Aguila et al. [99] examined transcriptomics in VTA and SNpc dopamine neurons in 18 human postmortem brains from individuals without neurological disease. In their study, the most highly enriched pathways in the VTA included ECM modulation, cytoskeletal regulation, and synapse formation, among several others. These related pathways were frequently among the top deregulated sets in the studies reviewed here and suggest that coordinated alterations in synaptic morphology and structure may be more damaging in the SNpc than in the VTA.

The GSEA findings described in this review exemplify the utility of omics studies, which provide an unbiased analysis of genetic variation and protein dysregulation, pointing to understudied pathways in disease. ECM and related pathways such as focal and cell adhesion are essential for neuronal development and structure, synaptic function, and cell survival [100,101,102,103]. However, the roles of ECM-related pathways and components remain understudied in PD and possibly other neurodegenerative diseases. In PD, synaptic dysfunction is an early pathological event that precedes neurodegeneration [91,104,105,106]. Given that ECM-related pathways are critical for synaptic function, stability, and plasticity [107], they may be targets for early intervention in PD.

The question then becomes which molecules to target, given that the genomic, transcriptomic, and proteomic alterations are present throughout ECM-related pathways. The answer to this question will be informed by further research into specific molecules and pathways and may involve cocktails that target multiple ECM-related molecules. Given their role as effectors, kinases are frequent therapeutic targets and have been pursued in PD [108]. Indeed, mutations in leucine repeat rich kinase-2 (LRRK2) are common risk factors for PD, and an LRRK2 inhibitor is in late-stage PD clinical trials [109]. However, kinases related more directly to the ECM and cell adhesion such as focal adhesion kinase have not been explored and may be potential targets if selectivity for affected brain regions could be achieved. Individual ECM-related molecules that may be potential therapeutic targets are suggested by various studies described earlier, including heparin and CD11b integrin. The intersection of ECM, cytoskeleton, synapse integrity, and synaptic vesicle recycling appears repeatedly in PD studies [110] and differentiates the VTA and SN [99], implying that cross-pathway research into synaptic dysfunction may provide clues to PD etiopathology and, ultimately, treatment. A related line of research involves the detachment of neurons from the ECM, which leads to cell death via anoikis—a type of programmed cell death. A recent diagnostic model of PD has been developed based on the differential expression of anoikis-related genes in human blood, implicating detachment from the ECM as an important factor in PD and suggesting an intervention point [111].

The present review has several limitations that are important to note. First, in most of the studies reviewed here, many other pathways than those related to the ECM were enriched. Indeed, the interpretation of omics pathway studies can be complex given the variety and sometimes apparently unrelated pathways identified as significant. Second, this review excluded studies that did not report significant ECM-related findings, as per the search criteria. A subsequent exploratory literature search designed to identify omics studies in PD that did not identify ECM-related pathways as top hits resulted in more than 20 studies [45,112,113,114,115,116,117,118,119,120,121,122,123,124,125,126,127,128,129,130]. However, upon closer review of these studies, it was noted that some limited the proteins or pathways that were included in the final models/reports for various reasons and, therefore, may not be expected to find ECM alterations (e.g., removal of genes expressed at low levels [112], inclusion limited to pathways with established relevance to PD [117]). Several other excluded studies reported significant findings for one or more individual ECM components rather than as part of a gene set [45,114,115] or as a significant interaction pathway rather than as part of a primary altered pathway [126]. Thus, although a substantial number of published omics studies do not report ECM-related pathways as significant, there is still ample reason to suspect their involvement and alteration.

An additional limitation of the present review relates to the small sample sizes of some of the included studies. Aguila and colleagues describe the importance of determining adequate sample sizes and using large-enough cohorts to reliably identify relevant DEGs; for their transcriptomic analysis, the minimum sample size needed was N = 8 [99]. This review did not take into consideration sample size, which could have led to spurious findings in some of the studies. Thus, caution is warranted in interpreting results from studies based on small sample sizes [99]. A related caution is the implicit assumption that gene sets/pathways with the lowest *p* values are the most important to the cell. Ranking gene sets according to *p* values does not necessarily correspond to the magnitude of their effects in the cell or their contribution to disease. These limitations underscore the need to complement findings from omics and bioinformatics analyses with in vitro and in vivo research. Based on the omics studies reviewed here, ECM-related pathways seem to be excellent candidates for such work.

## Figures and Tables

**Table 1:** Summary of included studies.

Authors, Year	Tissue	Method
Transcriptomics Studies		
Rosh et al., 2024 [26]	iPSC-derived DA neurons	RNA sequencing
Akrioti et al., 2022 [27]	iPSC-derived neural progenitor cells	RNA sequencing
Stern et al., 2022 [28]	iPSC-derived DA neurons	RNA sequencing
Hemmings et al., 2022 [29]	Blood	RNA sequencing
Cho et al., 2021 [30]	MSCs from adipose tissue	RNA sequencing
Fernandez-Santiago et al., 2021 [17]	Dermal fibroblasts	RNA sequencing
Yang et al., 2021 [31]	Blood	RNA sequencing
Booth et al., 2019 [32]	iPSC-derived, midbrain-patterned astrocytes generated from skin	RNA sequencing
Gonzalez-Cascuberta et al., 2018 [33]	Dermal fibroblasts	RNA sequencing
Tan et al., 2018 [34]	Blood	Microarray
Infante et al., 2016 [35]	Whole blood	RNA sequencing
Riley et al., 2014 [36]	Substantia nigra, striatum, cortex	Microarray
Durrenberger et al., 2012 [37]	Substantia nigra	Microarray
Zhang et al., 2012 [38]	Substantia nigra	Microarray
Edwards et al., 2011 [39]	Dorsal motor nucleus of vagus, locus coeruleus, substantia nigra, putamen, insula	Microarray
Grunblatt et al., 2004 [40]; Mandel et al., 2005 [41]	Substantia nigra, pars compacta	Microarray
Proteomics studies		
Bogetofte et al., 2023 [42]	iPSC-derived DA neurons	LC/MS
Jang et al., 2023 [43]	Substantia nigra	LC/MS
Acera et al., 2022 [44]	Tears	LC/MS
Zafar et al., 2022 [45]	Cerebrospinal fluid	LC/MS
Zhao et al., 2022 [46]	Plasma	LC/MS
Raghunathan et al., 2020 [18]	Prefrontal cortex	LC/MS
Jiang et al., 2019 [47]	Serum exosomes	LC/MS
Riley et al., 2014 [36]	Striatum, cortex	LC/MS
Genomics studies		
Sandor et al., 2017 [48]	Not reported	Whole exome sequencing
Hu et al., 2016 [49]	White blood cells	GWAS
Edwards et al., 2011 [39]	Not reported	GWAS
O’Dushlaine et al., 2009 [50]	Not reported	GWAS/SNP ratio test

Abbreviations: DA = dopamine, GWAS = genome wide association study, iPSC = induced pluripotential stem cells, LC/MS = liquid chromatography/mass spectrometry, MSC = mesenchymal stem cells, SNP = single nucleotide polymorphism.

## Data Availability

The data supporting the findings of this article are available within the article and its Supplementary Material.

## Supplementary Materials

The following supporting information can be downloaded at: https://www.mdpi.com/article/10.3390/brainsci14060522/s1. Table S1: Data extracted from studies included in this article.
